# Biomarkers for Health Functional Foods in Metabolic Dysfunction-Associated Steatotic Liver Disorder (MASLD) Prevention: An Integrative Analysis of Network Pharmacology, Gut Microbiota, and Multi-Omics

**DOI:** 10.3390/nu16183061

**Published:** 2024-09-11

**Authors:** Heng Yuan, Eun-Soo Jung, Soo-Wan Chae, Su-Jin Jung, James W. Daily, Sunmin Park

**Affiliations:** 1 Department of Bioconvergence, Hoseo University, Asan 31499, Republic of Korea; yuanheng.changan@gmail.com; 2 Clinical Trial Center for Functional Foods, Biomedical Research Institute, Jeonbuk National University Hospital, Jeonju 54907, Republic of Korea; esjung@jbctc.org (E.-S.J.); swchae@jbctc.org (S.-W.C.); sjjeong@jbctc.org (S.-J.J.); 3 Clinical Trial Center for K-FOOD Microbiome, Biomedical Research Institute, Jeonbuk National University Hospital, Jeonju 54907, Republic of Korea; 4 Research Institute of Clinical Medicine, Jeonbuk National University, Jeonju 54907, Republic of Korea; 5 Department of R&D, Daily Manufacturing Inc., Rockwell, NC 28138, USA; jdaily3@yahoo.com; 6 Department of Food and Nutrition, Obesity/Diabetes Research Center, Hoseo University, 20 Hoseoro79bungil, Asan 31499, Republic of Korea

**Keywords:** metabolic dysfunction-associated steatotic liver disease, HFFs, gut microbiota, omics approach

## Abstract

Metabolic dysfunction-associated steatotic liver disorder (MASLD) is increasingly prevalent globally, highlighting the need for preventive strategies and early interventions. This comprehensive review explores the potential of health functional foods (HFFs) to maintain healthy liver function and prevent MASLD through an integrative analysis of network pharmacology, gut microbiota, and multi-omics approaches. We first examined the biomarkers associated with MASLD, emphasizing the complex interplay of genetic, environmental, and lifestyle factors. We then applied network pharmacology to identify food components with potential beneficial effects on liver health and metabolic function, elucidating their action mechanisms. This review identifies and evaluates strategies for halting or reversing the development of steatotic liver disease in the early stages, as well as biomarkers that can evaluate the success or failure of such strategies. The crucial role of the gut microbiota and its metabolites for MASLD prevention and metabolic homeostasis is discussed. We also cover state-of-the-art omics approaches, including transcriptomics, metabolomics, and integrated multi-omics analyses, in research on preventing MASLD. These advanced technologies provide deeper insights into physiological mechanisms and potential biomarkers for HFF development. The review concludes by proposing an integrated approach for developing HFFs targeting MASLD prevention, considering the Korean regulatory framework. We outline future research directions that bridge the gap between basic science and practical applications in health functional food development. This narrative review provides a foundation for researchers and food industry professionals interested in developing HFFs to support liver health. Emphasis is placed on maintaining metabolic balance and focusing on prevention and early-stage intervention strategies.

## 1. Introduction

Metabolic dysfunction-associated steatotic liver disease, formerly known as non-alcoholic fatty liver disease (NAFLD), has emerged as a significant global health concern. The nomenclature change, officially announced in 2023, reflects a more accurate description of the etiology and pathophysiology of the disease [1]. This narrative review focuses on the early stages before the disease fully develops, which we term metabolic dysfunction-associated steatotic liver disorder (MASLD). MASLD refers to a spectrum of liver conditions associated with metabolic dysfunction, characterized by excessive fat accumulation in the liver without significant alcohol consumption (generally ≥ 30 g/day for men and ≥20 g/day for women) but which has not yet progressed to the disease state. It is closely linked to metabolic abnormalities, including hyperglycemia, hyperlipidemia, hypertension, and abdominal obesity [1]. 

Our review emphasizes MASLD as a precursor state, as functional foods are primarily associated with preventing or alleviating this condition rather than treating the advanced disease. This focus aligns with the role of functional foods in supporting health and potentially mitigating risk factors before the onset of more severe liver dysfunction or progression to disease. Over 5% fat in the liver is considered to be steatotic. The prevalence of metabolic-associated liver conditions has steadily increased worldwide, from 22% of the adult population in 1991 to 37% in 2019 [2], with notable differences among ethnic groups. The prevalence is highest among Hispanics, followed by Asians, Caucasians, and Africans [2]. A study predicts that by 2030, the global prevalence of MASLD will increase to 46.1% in males and 41.0% in females, while the prevalence of MASH will rise to 18.9% in males and 18.4% in females [3].

MASLD can have significant clinical consequences, particularly if it progresses to more severe forms. One major concern is the development of liver cirrhosis, which occurs when chronic liver inflammation and fat accumulation cause extensive fibrosis, impairing liver function [4]. Cirrhosis associated with MASLD increases the risk of complications such as liver failure, portal hypertension, and hepatic encephalopathy. The progression from MASLD to cirrhosis and eventually hepatocellular carcinoma underscores the importance of early diagnosis and management to prevent these serious outcomes [5].

The factors contributing to MASLD are multifaceted, involving a complex interplay of genetic predisposition, environmental influences, and lifestyle choices. Key elements in its development include metabolic dysfunction characterized by insulin resistance (IR), obesity, and components of metabolic syndrome (MetS) [6]. These conditions are associated with alterations in lipid metabolism, such as increased lipid influx into the liver, enhanced de novo lipogenesis, and impaired fatty acid oxidation [7]. At the MASLD stage, there may be early signs of cellular stress, including mild mitochondrial dysfunction and endoplasmic reticulum (ER) stress, which have not yet reached pathological levels [2]. Low-grade inflammatory processes and oxidative stress are often present, potentially setting the stage for liver damage if left unchecked. Additionally, gut microbiome compositional and functional changes may influence liver health. Genetic factors, such as variants in the patatin-like phospholipase domain-containing protein 3 (*PNPLA3*) gene, can increase susceptibility to liver fat accumulation [8]. These factors collectively create an environment conducive to liver fat accumulation and metabolic disturbances. However, at the MASLD stage, these processes are often reversible with appropriate lifestyle interventions and preventive measures, highlighting the importance of early detection and intervention.

Health functional foods (HFFs) are scientifically formulated products that offer specific health benefits beyond basic nutrition, often containing bioactive compounds from botanical sources such as polyphenols, alkaloids, saponins, and flavonoids [9]. Recent advancements in HFF research have been multifaceted and promising. The application of omics technologies has provided deeper insights into the mechanisms of action of functional ingredients [10]. The integration of gut microbiota research has led to the development of targeted prebiotic and probiotic foods [11]. Nanotechnology has improved the bioavailability and delivery of bioactive compounds [12], while the emerging field of personalized nutrition enables more tailored functional food solutions [13]. Furthermore, a growing focus is on sustainable sourcing and plant-based alternatives, aligning with consumer demands for environmentally friendly options [14]. These developments collectively demonstrate the evolving nature of HFF research and its potential to impact human health and nutrition.

To elucidate the complex mechanisms underlying the relationship between HFFs and metabolic dysfunction-associated steatotic liver disease (MASLD) prevention, researchers are employing advanced technologies such as network pharmacology and multi-omics approaches [9]. These methods provide a comprehensive view of the multi-component, multi-target nature of HFFs and their effects on liver health and metabolism. Recent in vivo studies have utilized integrative multi-omics analysis [10], combining metabolomics and transcriptomics, to reveal potential mechanisms by which certain HFFs, such as Alisma, Atractylodes, and milk thistle, prevent and/or alleviate liver damage and improve lipid metabolism. Transcriptomics offers insights into gene expression changes, while metabolomics reflects real-time dynamic changes in endogenous metabolites following intervention. This multi-omics approach has helped explain the relationship between the mechanisms of action and functions of these HFFs, supporting their development as therapeutic candidates for complex diseases like MASLD. By employing these advanced technological approaches, researchers are gaining unprecedented insights into how HFFs can prevent or alleviate MASLD. These advancements are crucial for developing more effective nutritional strategies and potential therapeutic interventions.

These cutting-edge methodologies promise to improve the understanding of MASLD etiology and identify and validate potential functional foods and their bioactive compounds that may positively impact MASLD progression. While foods are inherently complex mixtures of various compounds, this review focuses on identifying major food components that can modulate the multiple primary pathways involved in MASLD development. By targeting two to three key pathways simultaneously rather than focusing on a single mechanism [7], a more comprehensive strategy for designing effective functional foods could be developed for MASLD prevention and early intervention. This approach addresses the metabolic dysfunctions associated with MASLD before they progress to more severe liver conditions.

## 2. Identification of Biomarkers Associated with MAFLD 

We identified the biomarkers associated with the pathophysiological processes in MASLD that can be assessed in humans and validated in animal and cell models. Biomarkers linked to metabolomics, transcriptomics, and microbiomes were identified. Many physiological biomarkers directly indicate the presence of disease, whereas most microbiome biomarkers are more indirect and suggest the development of the disease. However, these indirect biomarkers are part of the processes involved in the etiology and progression of the disease. Once identified, the biomarkers could be used to generate data describing how various potential therapeutic agents interact with the pathophysiological processes associated with MASLD disease progression and regression. 

Histological analysis, such as liver biopsy, is considered the gold standard for diagnosing MASLD [15]. It remains the only technique that can reliably distinguish between simple liver steatosis and more advanced fibrotic disease [15]. Non-invasive techniques such as imaging techniques can identify the presence of fatty liver disease, but they cannot accurately determine the extent of disease progression to more advanced stages. In regular medical check-ups, common imaging methods include ultrasound, CT scans, and Magnetic Resonance Imaging Proton Density Fat Fraction (MRI-PDFF). Among these, MRI-PDFF is the most accurate non-invasive technique, providing precise quantification of liver fat content [16]. Because MASLD is so common, it should be suspected in anyone with abdominal obesity or impaired insulin sensitivity (diabetes or pre-diabetes) [11]. Abnormal liver function test results can indicate an increased likelihood of MASLD. These tests include the transaminase enzyme biomarkers, namely alkaline phosphatase (ALP), alanine transaminase (ALT), aspartate transaminase (AST), and gamma-glutamyl transferase (GGT) [12]. However, elevated levels of these liver enzymes are only suggestive of MASLD. While abnormal levels are seen in MASLD, they may also be elevated by factors other than MASLD [13]. Therefore, a diagnosis cannot be made based on the elevated levels of any one enzyme. A better way to utilize the enzyme biomarkers is to check the levels of all the enzymes: ALP, AST, the AST/ALT ratio (normal value = 1), and GGT. When the AST/ALT ratio is higher, it suggests liver damage. However, it could also be a result of muscle damage, and the ratio may remain elevated for as long as a week after heavy muscular exercise [14]. 

In diagnosing and assessing MASLD, various scoring systems and indices have been developed, combining serum biomarkers and imaging modalities. The Fatty Liver Index (FLI), a widely used non-invasive tool, incorporates body mass index, waist circumference, triglycerides, and gamma-glutamyl transferase to predict hepatic steatosis [1]. It is calculated using the formula: FLI = (e^0.953 × loge (triglycerides)^ + 0.139 × BMI + 0.718 × loge (GGT) + 0.053 × waist circumference − 15.745)/(1 + e^0.953 × loge (triglycerides)^ + 0.139 × BMI + 0.718 × loge (GGT) + 0.053 × waist circumference − 15.745) × 100 [17]. FLI scores range from 0 to 100, with higher scores indicating a greater likelihood of fatty liver. Other scoring systems include the Hepatic Steatosis Index (HSI = 8 × ALT/AST ratio + BMI + 2 if diabetes, + 2 if female) and the NAFLD Liver Fat Score (based on metabolic syndrome, type 2 diabetes, fasting insulin, AST, and ALT) [18]. The NAFLD fibrosis score combines age, BMI, diabetes status, AST/ALT ratio, platelet count, and albumin to assess fibrosis risk [15]. These non-invasive methods, often combined with imaging techniques like ultrasound or MRI-PDFF, offer valuable alternatives to liver biopsy for MASLD diagnosis and monitoring, enabling earlier detection and more effective disease management [15]. 

There is accumulating evidence supporting the association between elevated uric acid concentrations and MASLD [19], though the pathological mechanism is not yet clear. Preliminary evidence suggests that hepatic inflammation mediated through the NOD-like receptor protein 3 (NLRP3) inflammasome could be one of the mechanisms [20]. Another study indicates that MASLD is closely linked to the metabolism of fructose and subsequent uric acid generation that mediates fat accumulation [21]. The physiological biomarkers that indicate potential steatotic liver disease are shown in Table 1.

## 3. MASLD Pathogenesis

The primary risk factors for MASLD include obesity, type 2 diabetes, hyperlipidemia, and metabolic syndrome [27]. However, recent studies showed that non-obese individuals can also develop MASLD [28]. Approximately 10–20% of NAFLD patients had a relatively normal body mass index (BMI), a condition often referred to as non-obese or lean NAFLD. The pathophysiological mechanisms of MASLD primarily involve the abnormal accumulation of triglycerides (TG) in hepatocytes [29], which is closely associated with IR [30].

These changes can induce IR, a key factor in MASLD pathogenesis. IR is characterized by reduced insulin sensitivity, decreased cellular glucose uptake, increased insulin secretion, and exacerbated hyperinsulinemia, which further worsens hepatic IR. Insulin inhibits lipolysis in adipocytes under normal conditions [31,32]. In IR, inhibition of lipolysis is reduced, causing excessive free fatty acids (FFA) to flow into the liver and muscle, increasing hepatic lipid synthesis and lipid deposition, which further exacerbates IR [33].

Four different mechanisms regulate hepatic lipid accumulation: (1) increased hepatic uptake of circulating fatty acids, (2) increased hepatic de novo fatty acid synthesis, (3) decreased hepatic beta-oxidation, and (4) decreased hepatic lipid export. TG is synthesized from acyl-coenzyme A (CoA), and its concentration in hepatocytes depends on the level of FFA [34]. Approximately 60% of hepatic TG content originates from FFA released from the adipose tissue [35]. When FFA is excessive or very low-density lipoprotein (VLDL) secretion from the liver is impaired, there is an accumulation of TG in the liver. Excess FFA induces not only liver dysfunction but also causes damage to pancreatic β-cells, promoting apoptosis [34,36]. In MASLD patients with insulin-induced suppression of endogenous glucose production, VLDL-TG secretion is impaired [37].

Recent research has highlighted the role of the gut–liver axis in MASLD pathogenesis. The vagus nerve, part of the parasympathetic nervous system (PSNS), connects the gut microbiota and liver. The gut epithelium is a natural barrier, preventing harmful bacteria and elements from translocating into the circulatory system. MetS is associated with gut microbiota dysbiosis, which can damage the intestinal barrier and tight junctions, increase gut permeability, allow harmful substances to reach the liver via the portal vein, and induce inflammatory responses [38,39]. In summary, the MASLD etiology involves a complex interplay of lipid metabolism disorders, IR, and gut microbiota dysbiosis. These factors collectively contribute to excessive TG accumulation in hepatocytes, increased FFA influx, and inflammation. Understanding these mechanisms is crucial for developing effective MASLD prevention and treatment strategies.

## 4. Impact of Food Components on MASLD: Application of Network Pharmacology 

HFFs have gained significant attention in recent years due to their potential to prevent or mitigate disease symptoms in their early stages. Unlike conventional drugs, which typically contain a single active ingredient, HFFs are a complex mixture of natural compounds derived from plant or animal sources. The health benefits of these foods often result from the combined action of multiple components rather than a single compound [40]. This complexity presents a unique challenge in studying the effects of HFFs. Traditional reductionist approaches, which focus on isolating and studying individual compounds, are often inadequate for understanding the comprehensive effects of these food products. Consequently, there is a growing need for more comprehensive methodologies that can capture the intricate interactions between the multiple bioactive compounds present in HFFs and their effects on biological systems [41].

Network pharmacology, a systems biology approach, has emerged as a powerful tool for addressing this challenge. This methodology aims to explain the effects of bioactive compounds on biological systems, making it particularly well-suited for studying HFFs. By integrating data from various sources and modeling complex interactions, network pharmacology provides a holistic perspective that can reveal synergistic effects and elucidate the underlying mechanisms of action [17]. Sinisan is studied for alleviating MASLD via network pharmacology, and its mechanism is investigated [18]. Chinese herbal remedies, including Chaihu, Baishao, Zhishi, and Gancao, were found to have potential efficacy for treating NAFLD; their mechanisms of action were identified by network pharmacology, and efficacies were validated in animal studies [18]. It is reasonable to apply network pharmacology to investigate the efficacy and mechanisms of HFFs. 

**Figure 1:**
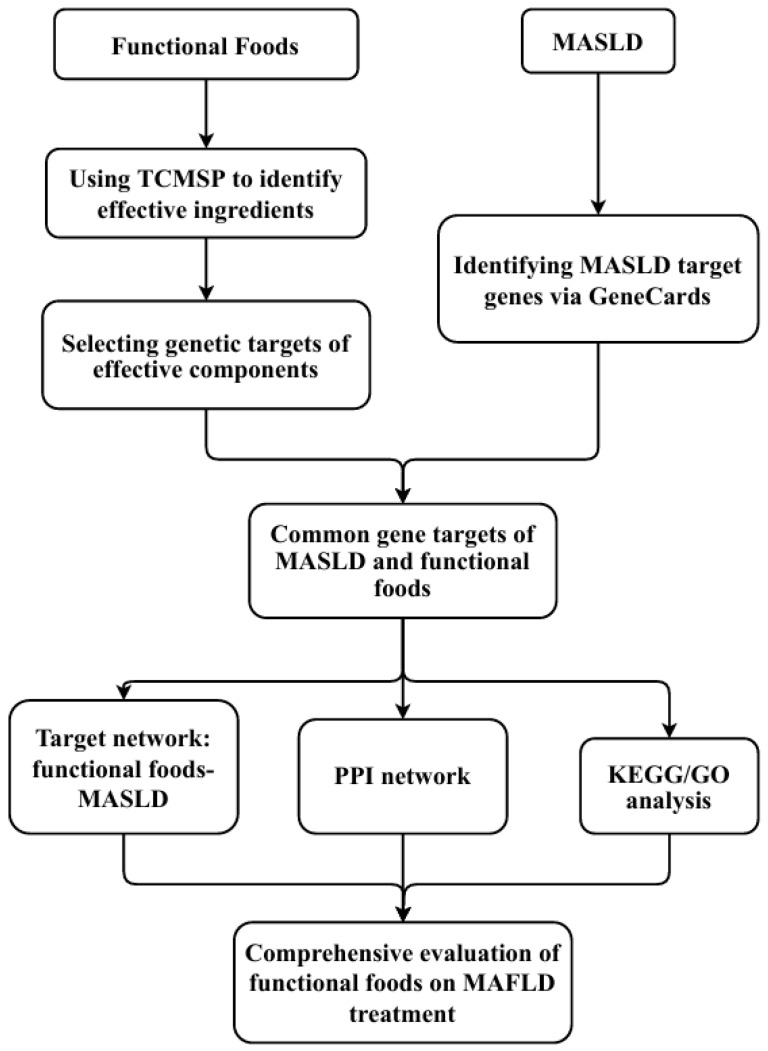
Flowchart of the research process for developing health functional foods (HFFs) to treat metabolic dysfunction-associated steatotic liver disorder (MASLD) using network pharmacology.

The research process for developing HFFs to treat MASLD using network pharmacology is illustrated in Figure 1. This approach typically begins with identifying bioactive compounds of interest in the HFFs. Bioactive compounds in foods can be found on several websites, such as FooDB and health functional food databases, or directly measured from the food sources by liquid chromatography-tandem mass spectrometry (LC-MS/MS) and ultra-performance liquid chromatography-quadrupole (UPLC-Q)-Exactive Orbitrap MS. Following the identification of the compound, targets are typically predicted using databases such as Swiss Target Prediction or Search Tool for Interacting Chemicals (STITCH), focusing on proteins known to be involved in MASLD pathogenesis, such as AKT serine/threonine kinase 1 (AKT1), interleukin-6 (IL6), vascular endothelial growth factor A (VEGFA), tumor necrosis factor (TNF), and peroxisome proliferator-activated receptor gamma (PPARG). Next, target metabolites (compounds) and proteins are used to construct a metabolite-target-disease network that can be used to predict potential mechanisms of HFFs against MASLD using tools like Cytoscape [42]. Further analysis involves constructing a protein–protein interaction (PPI) network to help understand the interactions between target proteins and other relevant proteins in the biological system. KEGG pathway analysis and Gene Ontology (GO) analysis are performed to identify the biological pathways and processes most likely to be affected by the compounds, helping to predict potential mechanisms of action at a systems level. In silico validation through molecular docking simulations can predict binding affinities between compounds and potential protein targets, providing computational validation of predicted interactions. Finally, key predictions from the network pharmacology analysis are validated through in vitro and in vivo experiments, potentially including cell-based assays, animal models, and clinical trials. 

Network pharmacology offers several advantages. These include its ability to provide a holistic perspective on complex biological interactions, efficient screening of potential therapeutic compounds, and prediction of synergistic effects among multiple components. This approach can significantly reduce the time and cost associated with traditional experimental methods by guiding researchers toward the most promising targets and pathways. Additionally, it allows for integrating diverse data types, from genomics to metabolomics, providing a more comprehensive understanding of disease mechanisms and potential interventions. However, network pharmacology also has limitations. The accuracy of its predictions heavily depends on the quality and completeness of the underlying databases, which may contain biases or gaps in information. There is also a risk of false positives due to the many potential interactions considered. Moreover, the approach may oversimplify complex biological systems, potentially missing subtle or context-dependent effects. Due to the computational nature of the method, the predictions will require experimental validation, and the transition from in silico predictions to in vivo effectiveness can be challenging. Finally, interpreting network pharmacology results requires expertise in both computational biology and the specific disease area, which may limit its accessibility to some researchers. Overall, this systematic research approach not only improves the efficiency of HFF discovery but also provides a scientific basis for developing new therapeutic strategies. 

## 5. Association of Gut Microbiota and Their Metabolites with MASLD

### 5.1. Association of Gut Microbiota with MASLD

Gut microbiota dysbiosis plays a crucial role in MASLD progression [43]. Cholesterol-induced MASLD is associated with microbiota dysbiosis, and inhibiting dysbiotic gut microbiota and related metabolites might be an effective MASLD prevention strategy [44]. Gut microbiota play important roles in the gut–liver axis, participating in normal host physiology, resisting exogenous pathogenic microbes through active and/or competitive mechanisms, and maintaining gut barrier integrity [45]. Harmful substances like microbe-associated molecular pattern (MAMP) and proadrenomedullin N-terminal 20 peptide (PAMP) potentially alter gut permeability to disrupt the gut barrier and enter into the liver via mesenteric and portal venous circulation, causing liver damage and systemic inflammation [46]. Dysbiosis alters the gut microbiome’s composition and function, potentially leading to changes in short-chain fatty acid (SCFA) production and composition. This imbalance can increase endotoxin generation, triggering Kupffer cell inflammation, disrupting bile acid enterohepatic circulation, and contributing to hepatic inflammation and steatosis. The altered SCFA profile may further impact metabolic processes and liver function [47]. Gut-derived metabolites regulate macrophage and hepatocyte inflammation [48]. Studies show that gut microbiota-produced lipopolysaccharides (LPS) and minor protein endotoxins exacerbate the hepatic load, causing hepatocyte damage mediated by Kupffer cells and inflammatory mediators [49]. In dysbiosis, there is an increase in Gram-negative bacteria, which produce large amounts of LPS that enter the liver, activating the Toll-like receptor 4 (TLR4) signaling pathway and worsening MASLD [50]. Su et al. have reported that polychlorinated biphenyl (PCB) induces gut dysbiosis in mice, leading to hepatic lipid accumulation and damage [51]. Additionally, gut microbiota can influence secondary bile acid metabolism, altering the balance of anti-inflammatory and pro-inflammatory cytokines secreted by M1 and M2 macrophages, and impacting the liver’s immune function [52].

Developing effective HFFs requires identifying specific types of beneficial and harmful bacteria. The beneficial effects of HFFs include anti-inflammatory actions within the gut and a favorable impact on metabolic parameters [53]. Mulberry and silk amino acids, ginseng, and Aronia have been demonstrated to alleviate liver damage and fat deposition via modulating gut microbiota [54,55,56]. Several studies have compared the gut microbiota composition in patients with MASLD and MASH to healthy controls. Two studies found an increased abundance of *Lactobacillus* and decreased levels of *Ruminococcaceae* in MASLD patients [57,58]. Another study observed a reduction in *Bacteroides* in MASH patients [59]. In children with MASH, a gradual increase in *Proteobacteria* was noted [60]. Furthermore, a study comparing obese and lean patients with and without MASH revealed that lean MASH patients had higher levels of *Faecalibacterium* and *Ruminococcus*, whereas obese MASH patients had an increased abundance of *Lactobacillus* compared to healthy individuals. Overall, Coprococcus, Eubacterium, and Lachinospiraceae decrease, and Adidaminococcus and Escherichia increase in MASLD patients compared to healthy persons [61]. 

HFFs should aim to promote the growth of beneficial bacteria while inhibiting the proliferation of harmful bacteria [62]. However, most studies on bacteria have been at the genus level, which poses challenges for precise modulation as many species within the same genus form complex, interacting, and networking clusters. To effectively prevent and mitigate MASLD and MASH, it is crucial to identify the specific bacterial species involved and understand their intricate network interactions. With this knowledge, HFFs can be strategically designed to modulate bacterial communities by elevating beneficial species using targeted bioactive compounds, prebiotics, or probiotics. This approach would allow for more precise and effective interventions to modulate gut microbiota composition, potentially leading to improved outcomes in metabolic liver diseases. 

### 5.2. Relationship between Gut Microbial Metabolites and MASLD

**Figure 2:**
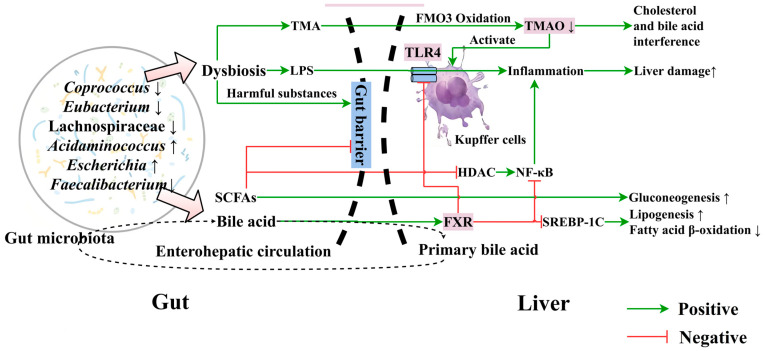
Gut microbiota and their metabolites in metabolic dysfunction-associated steatotic liver disorder (MASLD) progression. Schematic representation of gut microbiota dysbiosis and its impact on liver health in MASLD. Dysbiosis is characterized by a decreased abundance of Coprococcus, Eubacterium, Lachnospiraceae, and Faecalibacterium, with increased Acidaminococcus and Escherichia. This imbalance leads to elevated production of lipopolysaccharide (LPS) and trimethylamine (TMA). LPS activates Kupffer cells via Toll-like receptor 4 (TLR4), triggering the histone deacetylase (HDAC), nuclear factor kappa-light-chain-enhancer of activated B cells (NF-κB) pathway and causing inflammation. TMA is oxidized to trimethylamine N-oxide (TMAO) by flavin-containing monooxygenase 3 (FMO3), interfering with cholesterol and bile acid metabolism. Short-chain fatty acids (SCFAs) promote gluconeogenesis, whereas bile acids interact with the farnesoid X receptor (FXR) receptor, inhibiting sterol regulatory element binding protein 1c (SREBP-1c) and affecting lipogenesis and fatty acid β-oxidation. Green arrows indicate positive effects; red lines represent inhibitory actions. The dashed line shows the enterohepatic circulation of bile acids. This figure highlights the intricate relationship between gut microbiota, their metabolites, and liver function in MASLD progression. Up and down arrows indicated the increase and decrease of the function.

Microbial metabolites, resulting from complex microbe–microbe and host–microbe interactions, are increasingly recognized as integral to human physiology. These metabolites, including SCFAs and bile acids, profoundly influence immune function and dysfunction, playing significant roles in health and disease (Figure 2) [63,64]. 

#### 5.2.1. Bile Acids

The sizes and compositions of the bile acid pool and the gut microbiota community are intricately linked, with each significantly influencing the other. Maintaining a dynamic balance between these elements is crucial for overall health [65]. Under physiological conditions, bile acids play a role in lipid digestion and metabolic regulation [66]. Bile acids, particularly through the farnesoid X receptor (FXR) signaling pathway, are key modulators of lipid and glucose metabolism [67,68]. The activation of FXR has multiple beneficial effects: it suppresses nuclear factor kappa B (*NF-κB*), thereby reducing liver inflammation [68,69], and downregulates the transcription factor sterol regulatory element-binding protein (*SREBP-1c*), thereby decreasing lipogenesis and increasing β-oxidation of fatty acids [70]. Furthermore, bile acid signaling through FXR and G protein-coupled bile acid receptor 1 (*GPBAR1*) influences Toll-like receptor (TLR)-dependent pathways and NRLP3-dependent inflammasome activation, which are crucial in immune regulation [71,72]. However, the relationship between bile acids and MASLD is complex. Whereas FXR activation generally has protective effects [73], alterations in the bile acid pool, particularly the elevation of the levels of certain secondary bile acids, have been associated with MASLD progression [74]. These effects may be mediated through increased gut permeability, leading to endotoxemia and indirectly causing IR [75]. 

The gut microbiome plays a critical role in bile acid metabolism by deconjugating primary bile acids and converting them into secondary bile acids [76,77]. This microbial transformation of bile acids can influence MASLD development [78]. Conversely, changes in the bile acid pool can reshape the gut microbiota, creating a bidirectional relationship in the gut–liver axis [67]. Therefore, bile acids and the gut microbiome interplay is highly complex and bidirectional. While bile acids, through FXR and GPBAR1 signaling, can inhibit liver fat accumulation and inflammation, alterations in the bile acid pool and gut microbiota can also contribute to MASLD pathogenesis. This intricate relationship highlights the potential of bile acids as contributors to disease. It serves as therapeutic targets in MASLD, emphasizing the need for a nuanced understanding of their role in metabolic health.

#### 5.2.2. Short-Chain Fatty Acids (SCFAs)

SCFAs are vital mediators between the microbiota and the immune system and cellular targets essential for maintaining gut homeostasis, and they play crucial roles in MASLD development [79]. SCFAs, primarily acetate, propionate, and butyrate, are the main products of anaerobic bacterial fermentation of indigestible carbohydrates. Changes in carbohydrate consumption and dysbiosis can alter the types and amounts of SCFAs synthesized in the gut, potentially influencing MASLD progression through multiple mechanisms [76]. SCFAs produced in the gut, such as acetate and propionate, are energy sources for the liver, playing essential roles in hepatic lipogenesis and gluconeogenesis [80,81]. Propionate can reduce hepatic fat synthesis, whereas acetate serves as a substrate for lipogenesis, is a precursor for both cholesterol and fatty acids, and is involved in IR [82]. However, the relationship between SCFA and MASLD remains controversial.

SCFAs contribute to epithelial barrier protection, with acetate enhancing intestinal epithelial cell integrity and boosting resistance to infections. SCFA supplementation has been shown to protect mucosal immunity [83]. The mechanisms of SCFA action are diverse, and they function as inhibitors of histone deacetylases (HDACs) and as ligands for G protein-coupled receptors (GPCRs), particularly free fatty acid receptors 2 (FFAR-2, also known as GPR43) and FFAR-3 (GPR41) [84]. These interactions influence various immune cells and metabolic processes. For instance, the activation of GPR41 and GPR43 in adipocytes inhibits lipolysis and promotes adipocyte differentiation, while GPR43 activation in intestinal neutrophils can increase gut inflammation and permeability, potentially contributing to MASH [85].

The role of SCFAs as HDAC inhibitors may promote hepatic tolerance and anti-inflammatory cell phenotypes, helping to maintain immune homeostasis [86]. They inhibit NF-κB activity, downregulate pro-inflammatory cytokine production, and induce tolerogenic responses in various immune cells [87,88]. Their effects on neutrophils are particularly complex, as they enhance their activity and migration while inhibiting certain effector functions [86]. However, it is important to note that while SCFAs appear to have various effects on processes related to MASLD, currently, there is insufficient data to fully elaborate their specific roles for MASLD. The effects of SCFAs on MASLD are complex and may vary depending on the specific SCFA, its concentration, and the stage of the disease. More research is needed to clarify these relationships and their potential therapeutic implications. 

Developing HFFs that modulate gut microbiota and their metabolites is a promising approach for managing MASLD [73]. Foods containing probiotics, prebiotics, and dietary fibers can promote SCFA production, regulate immune responses and inflammation, and enhance gut and liver health [74]. These dietary interventions provide new directions and strategies for MASLD prevention and treatment. However, further research on the impact of bile acids and SCFAs on specific immune cell subsets and functions is crucial to fully understanding the immunoregulatory mechanisms of gut microbiota and their metabolites in MASLD [72]. This knowledge is essential for developing more effective interventions. The intricate interplay between microbial metabolites, the immune system, and metabolic processes underscores the importance of continued investigation in this field to combat this increasingly prevalent metabolic disorder.

#### 5.2.3. Trimethylamine (TMA) and Trimethylamine N-Oxide (TMAO)

In addition to SCFAs and bile acids, another important class of gut microbial metabolites linked to MASLD etiology is TMA and its oxidized form, TMAO. Certain gut bacteria produce TMA produced from dietary precursors such as choline, L-carnitine, and betaine [89]. Once absorbed, TMA is rapidly converted to TMAO by hepatic flavin-containing monooxygenases (FMOs) in the liver [90]. Elevated levels of TMAO have been associated with various metabolic disorders, including MASLD. The connection between TMAO and MASLD pathogenesis is multifaceted, involving increased inflammation, oxidative stress, and alterations in lipid and glucose metabolism [90]. Studies have shown that individuals with MASLD often exhibit higher serum TMAO levels compared to healthy controls, suggesting a potential role of this microbial metabolite in the development and progression of the condition [91]. The TMA/TMAO pathway represents an important example of how gut microbial metabolism can influence liver health, highlighting the complex interplay between diet, gut microbiota, and host metabolism in MASLD.

### 5.3. Effects of Food on Gut Microbiota Balance and the Gut–Liver Axis

**Figure 3:**
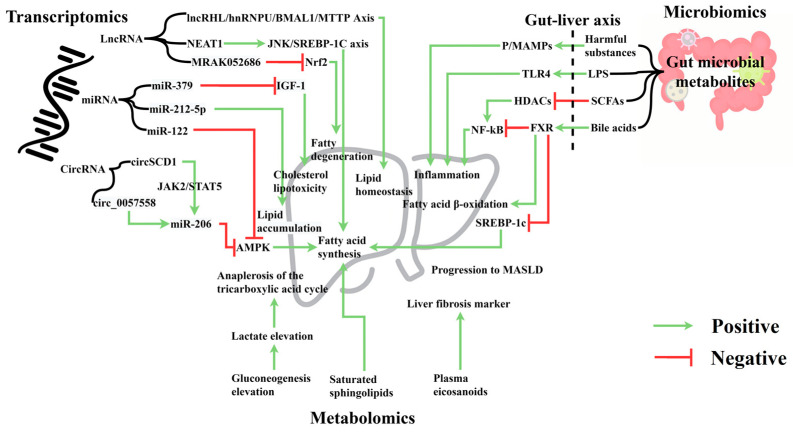
Transcriptomics, metabolomics, and microbiomics in metabolic dysfunction-associated steatotic liver disorder (MASLD). This figure illustrates the interplay between transcriptomics, metabolomics, and the gut–liver axis in MASLD etiology. Transcriptomics: lncRNAs (NEAT1, MRAK052686) promote fatty degeneration via c-Jun N-terminal kinases (JNK)/sterol regulatory element binding protein 1c (SREBP-1c) and nuclear factor erythroid-2-related factor 2 (Nrf2) pathways; miRNAs (miR-379, miR-212-5p, miR-122) negatively impact insulin-like growth factor (IGF)-1 signaling and AMP kinase (AMPK); circRNAs (circSCD1, circ_0057558) influence lipid metabolism through Janus Kinase2 (JAK2)/signal transducer and activator of transcription 5 (STAT5) and miR-206 pathways. Metabolomics: shows hepatic changes including tricarboxylic acid cycle anaplerosis, elevated lactate and gluconeogenesis, and synthesis of saturated sphingolipids and plasma eicosanoids. Gut–liver axis: microbial metabolites affect liver function via Toll-like receptor 4 (TLR4), histone deacetylases (HDACs), and farnesoid X receptor (FXR) pathways. Green arrows indicate positive regulatory effects; red arrows represent negative impacts. This figure demonstrates the complex molecular interactions underlying MASLD progression.

The gut–liver axis theory, proposed by Marshall in 1998, describes the complex network of communication between the gut microbiota and the liver via the portal vein, stemming from their close anatomical and physiological relationship [92]. The maintenance of the gut–liver axis balance is closely related to the gut microbiota composition, gut barrier, and bile acid metabolism, which complement each other [93] and are potentially involved in vagal nerve activation (Figure 3) [93].

When the gut microbiota composition is altered, there can be functional changes that compromise the gut mucosal barrier’s integrity, increasing permeability [94]. Bacteria and metabolites can reach the liver via the portal vein, interfering with bile acid metabolism, activating the liver’s innate immune system, releasing inflammatory factors and vasoactive substances, triggering oxidative and endoplasmic reticulum stress, and causing hepatocyte degeneration and apoptosis [95]. These metabolites can also damage the gut mucosa, creating a vicious cycle closely related to MASLD pathogenesis and affecting prognosis [95].

Tryptophan-derived microbial metabolites are important signaling molecules for host–microbe communication, potentially maintaining gut and systemic homeostasis. Serotonin (5-HT), derived from tryptophan metabolism, regulates various organs through 5-HT receptors (5-HTR). When 5-HT binds to liver 5-HTR, it lowers the levels of tumor necrosis factor-α (TNF-α) and NF-κB, modulating hepatic macrophage immunity, inhibiting inflammation, and protecting the liver from multiple insults, making this an important MASLD prevention mechanism [96,97].

Probiotics, polyphenols, natural polysaccharides, oligosaccharides, and vitamins can mitigate MASLD by improving gut microbiota homeostasis, protecting the gut barrier, inhibiting PAMP translocation, reducing oxidative stress, and decreasing inflammation infiltration [98,99]. These substances are commonly found in easily accessible fruits, vegetables, and fermented foods. Thus, targeting the gut–liver axis for nutritional intervention may be a crucial strategy for improving the health of MASLD patients.

### 5.4. Potential of Modulating the Gut Microbiota to Improve MASLD

Microbiome-targeted therapies (MTT) use HFFs, probiotics, prebiotics, and antibiotics to correct or rebuild gut microbiota in the treatment of dysbiosis and other diseases [100]. Supplementation with dietary fibers like lactulose, oligofructose, and inulin stimulates gastrointestinal peptide release, regulating appetite and energy metabolism [101]. However, excessive inulin intake in TLR5-deficient mice raised bilirubin levels, indicating potential liver damage from high inulin intake. This preliminary study in an animal model highlights the need for medical guidance in specific types of fiber supplementation to eliminate harmful bacteria [102]. Fecal microbiota transplantation (FMT) is a comprehensive method to restore healthy microbiota composition. It has been demonstrated to be an effective therapy for *Clostridium difficile*-associated diarrhea, pseudomembranous colitis, and chronic inflammatory bowel diseases like ulcerative colitis and Crohn’s disease [103]. Prebiotic therapy has been shown to correct gut dysbiosis in high-fat diet mice, increasing the levels of the tight junction protein zonula occludens-1 (ZO-1) and alleviating MASH [104]. Commercial probiotics like *Streptococcus*, *Lactobacillus*, and *Bifidobacterium* promote anti-inflammatory environments, supporting intestinal epithelial cell growth and survival, and combating pathogens through immune system regulation and host defense [105]. *Lactobacillus* and *Bifidobacterium*, as safe probiotics, can lower blood cholesterol [106].

## 6. Omics Approaches in MASLD Research

### 6.1. Transcriptomics and MASLD

Transcriptomics quantifies coding and non-coding RNA transcripts, reflecting cellular transcriptional activity [107]. MASLD transcriptomics links genetic information with steatosis, inflammation, and fibrosis protein profiles, offering insights into the gene regulation mechanisms and biological processes in MASLD [108]. Research shows that nuclear receptor subfamily 2 group F member 6 (NR2F6) promotes MASLD by activating *CD36* gene expression, inducing TG retention, and providing a new molecular basis for hepatic steatosis [109]. Global liver RNA profiling identified 535 long non-coding RNAs (lncRNA) and 760 mRNAs overexpressed in MASLD [110]. Cohort studies involving liver and blood lncRNA and circular RNA (circRNA) expression could further validate the clinical value of transcriptomics.

#### 6.1.1. Micro RNA (miRNA)

miRNAs are endogenous non-coding RNAs that regulate post-transcriptional gene expression and play crucial roles in various biological processes [111]. miRNA binding sites in closed circular nucleotide chains form circRNA-miR-mRNA axes or networks; inhibiting or promoting related target gene expression; and participating in MASLD progression. For instance, miR-122 upregulation and sirtuin 1 (*SIRT1*) downregulation inhibit the AMP-dependent protein kinase (*AMPK*) pathway, promoting lipogenesis [112]. Next-generation RNA sequencing of miRNA expression in liver biopsies of grade III obese patients showed elevated miR-301a-3p and miR-34a-5p and reduced miR-375, promoting MASLD progression to hepatocellular carcinoma [113]. Real-time PCR analysis of miRNA levels of a MASLD patient revealed that miR-212-5p downregulation promotes lipid accumulation [114]. Serum miR-379 increases cholesterol lipotoxicity by interfering with insulin-like growth factor 1 signaling [115]. Circulating miR-21 levels and their hepatic expression can increase in MASLD patients and mouse models of MASLD [116]. Thus, miRNAs are potential therapeutic targets that offer new strategies for MASLD clinical treatment.

#### 6.1.2. lncRNA

lncRNAs, non-coding RNAs over 200 nucleotides long, regulate transcription of protein-coding genes. lncRNAs may be diagnostic and therapeutic targets for MASLD. Genetic studies in mice and primary hepatocytes show that lncRNA regulator of hyperlipidemia (lncRHL) activates the lncRHL/heterogeneous nuclear ribonucleoprotein U (hnRNPU)/brain and muscle aryl hydrocarbon receptor nuclear translocator (ARNT)-like protein 1 (BMAL1)/microsomal triglyceride transfer protein (MTTP) axis, revealing new molecular mechanisms of lipid homeostasis in the liver and blood circulation [117]. lncRNA binds hnRNPU, transcriptionally activating BMAL1, thereby inhibiting VLDL secretion from hepatocytes. lncRNA nuclear-enriched abundant transcript 1 (NEAT1) exacerbates FFA-induced hepatic lipid accumulation by regulating the c-Jun N-terminal kinase (JNK)/SREBP-1c axis. Sorafenib resistance-associated lncRNA (lncARSR) levels are increased in the serum and liver of MASLD patients [118]. lncRNA (MRAK052686) correlates with antioxidant factor Nrf2, and its downregulation promotes steatosis [119]. Evidence suggests that MASLD development is associated with abnormal lncRNA expression.

#### 6.1.3. circRNA

CircRNAs, non-coding RNAs related to lipid metabolism, are potential therapeutic targets for liver diseases. High-fat diet mice show aberrant CircStearoyl CoA desaturase 1 (circSCD1) expression, affecting the degree of steatosis and promoting MASLD via the JAK2/STAT5 pathway [120]. Bioinformatic modeling shows genome-scale circRNA dysregulation associated with hepatic steatosis [121]. In MASLD mice and in vitro cells, circ_0057558 regulates Rho-associated protein kinase 1/AMPK signaling by targeting miR-206, promoting MASLD [122]. CircRNA dysregulation is closely linked to MASLD.

### 6.2. Metabolomics and MASLD

Metabolomics analyzes endogenous low-molecular-weight metabolites in various biological samples, helps monitor multiple metabolic pathways, and is useful for studying MASLD. Saturated sphingolipids significantly correlate with visceral fat in non-obese MASLD, accompanied by enhanced gluconeogenesis, lactate production, and the tricarboxylic acid cycle. Acetate and propionate supplementations reduce hepatic lipid accumulation and inflammation, inhibiting cholesterol synthesis via the AMPK and acetyl-CoA carboxylase (ACC) pathways, reducing TNF expression, and hepatic fatty acid synthase activity [123,124]. Blood sample studies indicate that serum S-adenosylhomocysteine, homocysteine, and plasma protein C levels may positively correlate with MASLD prevalence [125]. Plasma eicosanoids are biomarkers for hepatic fibrosis in MASLD patients [126], providing new strategies for timely detection and intervention in MASLD. The levels of microbial metabolites specific to MASLD increase with its progression [127]. Besides blood metabolomics, exploring the correlation between MASLD staging, urinary metabolomics, and intermediate metabolites can help identify new parameters and determine metabolic differences across MASLD stages to develop effective and specific treatments [128,129]. Integrating metabolomics data and clinical information helps to explain the molecular characteristics of MASLD, assists in identifying at-risk patients, and provides markers for personalized medicine in MASLD [130].

### 6.3. Importance of Integrated Multi-Omics Analysis

In contemporary medical research, integrating diverse methodologies has become crucial for unraveling disease mechanisms and developing novel therapies. Network pharmacology, gut microbiome research, and multi-omics data integration offer innovative perspectives and approaches for studying MASLD. By comprehensively utilizing these methods, researchers can gain a more holistic understanding of the disease’s complex mechanisms, identify new therapeutic targets, and develop more effective intervention strategies. 

A recent study exemplified this approach by investigating the therapeutic effects of secondary metabolites from oats (*Avena sativa*) and gut microbiota on MASLD through network pharmacology. The methodology encompassed database screening of metabolites, identification of relevant targets, construction of PPI networks, and molecular docking analysis with computational validation. The results revealed strong binding affinities between specific compounds and targets: myricetin and quercetin from gut microbiota with *VEGFA*, diosgenin with *IL-2*, and vestitol from oats with *NR4A1*. These findings suggested potential therapeutic effects on MASLD through regulation of the PI3K-Akt signaling pathway, underscoring the importance of dietary strategies and beneficial gut microbiota in MASLD management [115]. 

Similarly, Oh et al. employed network pharmacology to screen and test natural flavonoids (DPDNFs) derived from the diet and gut microbiota, uncovering potential MASLD therapeutic effects. Their research showed that flavonoids such as quercetin and myricetin acted on key targets including *AKT1*, *CFTR,* and PIK3 regulatory subunit 1 (*PIK3R1*), inhibiting the cAMP signaling pathway in the liver and consequently reducing MASLD. The study also identified key microbes, including *Enterococcus* sp. 45, *Escherichia* sp. 12, and *Escherichia* sp. 33, which played crucial roles in MASLD treatment [116]. 

These studies highlight the power of combining network pharmacology, gut microbiome analysis, and multi-omics data in elucidating the complex mechanisms of MASLD and identifying novel therapeutic targets and strategies. Further research into the role of the gut microbiome and its metabolites in the etiology of MASLD could pave the way for developing more precise and effective functional foods and pharmacological interventions. Future studies should continue to explore the potential of these integrated approaches in MASLD treatment, with the ultimate goal of improving patients’ health outcomes and quality of life.

MASLD is a metabolic stress-related liver disease closely linked to obesity. MetS includes simple steatosis, MASH, and related cirrhosis and hepatocellular carcinoma [131]. Single-omics data only analyze the association of MASLD with a specific biochemical marker but cannot explain complex causal relationships. Advancements in genomics, transcriptomics, epigenomics, proteomics, and metabolomics have enabled integrated multi-omics data analysis. This approach can identify key regulatory pathways and biomarkers, providing insights into the pathogenic mechanisms and disease progression of MASLD. It also offers a theoretical foundation for discovering potential therapeutic targets, developing personalized medicine protocols, and strategies for the development of HFFs for MASLD intervention. This paper reviewed the progress in multi-omics research related to MASLD, providing a comprehensive theoretical basis and new strategies for its prevention and treatment.

## 7. Impact of the Korean HFF Regulatory Framework and Its Implications for MASLD Research

The development and marketing of HFFs in Korea are governed by stringent regulations overseen by the Ministry of Food and Drug Safety (MFDS). This rigorous regulatory framework ensures that HFFs meet high functionality, safety, and quality standards before being recognized for preventing or mitigating disease states, including early signs of MASLD. To obtain MFDS recognition, HFF candidates must undergo a comprehensive evaluation process that includes the following: (1) Health claims: This requires scientific evidence from in vitro studies, in vivo studies in animal models, and human clinical trials. The data must clearly show the food’s beneficial effects on health or for preventing MASLD. (2) Elucidation of the mechanism of action: Researchers should provide a detailed explanation of the biological mechanisms through which the HFF or functional ingredient exerts its effects. This often involves identifying active compounds and their molecular targets. (3) Safety assessment: Following the MFDS-established flowchart to determine whether a functional ingredient can be considered a food ingredient or if additional toxicological studies are required to ensure safety. This process helps decide whether the ingredient can be accepted as safe for using in HFF or if further safety data are needed (Figure S1).

This approach differs from regulations in other countries. The United States Food and Drug Administration (FDA) has relatively lenient requirements, focusing primarily on food safety and labeling accuracy [36]. The European Food Safety Authority (EFSA) demands scientific evidence to support health claims, but it has less detailed trial requirements than Korea. Japan’s Foods for Specified Health Uses (FOSHU) system imposes strict requirements on efficacy and safety but allows companies to voluntarily submit safety data [37]. The stringent recognition process in Korea has significant implications for MASLD research and the development of HFFs targeting this condition. It encourages the integration of new technologies, including genomics, proteomics, metabolomics, and gut microbiota analysis, into research strategies for MASLD-targeted HFFs. This approach can significantly enhance the quality and depth of evidence presented to the MFDS for health claims.

Ultimately, this regulatory framework drives high standards in MASLD research by mandating comprehensive scientific validation. It leads to an enhanced understanding of MASLD mechanisms and the development of more effective functional foods aimed at early prevention and intervention while ensuring product safety for consumers.

## 8. Future Research Pathways and Final Conclusions

This review highlights the significant potential of integrating transcriptomics, metabolomics, and microbiomics for screening HFFs, focusing on MASLD management. By combining these methods, researchers can gain a more comprehensive understanding of the complex mechanisms of MASLD, identify new therapeutic targets, and develop more effective intervention strategies. This approach ensures that HFF candidates are effective and scientifically validated, supporting new product development and promoting advancements in the field of HFFs. 

Future research should focus on implementing comprehensive microbiome analysis in health functional food screening, including gut microbiota profiling and microbial metabolite analysis. Developing more sophisticated host–microbe interaction studies and advancing multi-omics data integration techniques will be crucial for constructing comprehensive interaction networks and identifying key regulatory pathways. Combining network pharmacology with microbiomics can predict synergistic effects and elucidate complex mechanisms of action. Further investigation into the specific mechanisms by which HFFs affect MASLD, particularly through the modulation of the gut microbiome and related metabolites, is necessary. 

This integrated approach can establish a rapid screening method for functional compounds, laying the foundation for “function-formula-component” quality control. By combining these methods into a coherent screening strategy, researchers can ensure that HFF candidates are effective, safe, and tailored to individual metabolic profiles. The focus should be on further elucidating the role of the gut microbiome and its metabolites in MASLD, which could lead to developing more precise and effective HFFs and pharmacological interventions. This comprehensive approach holds promise for revolutionizing the development of HFFs, particularly for complex metabolic diseases like MASLD. It has broad application potential in personalized nutrition and medicine. Ultimately, these integrated approaches aim to improve health outcomes, the quality of life of patients, and our understanding and treatment of MASLD.

## Figures and Tables

**Table 1:** Physiological biomarkers for evaluating the effects of health functional foods on MASLD.

Name	Normal Values	Description
Percent body fat	≤25.8% men ≤37.1% women	Body fat percent greater than the reference range increases the risk of cardiovascular and associated diseases.
Hemoglobin A1c (HbA1c)	<5.7%	Elevated levels indicate pre-diabetes or diabetes, depending on the level of elevation. It is a major risk factor for MASLD.
Alanine transaminase (ALT)	7–56 IU/L	Elevation usually indicates liver damage but is not always indicative of liver injury alone.
Aspartate transaminase (AST)	0–35 IU/L	Less specific to the liver than ALT and can reflect damage in many tissues, including the liver.
AST/ALT ratio	1 (1:1)	A higher or lower ratio is a better indicator of liver damage than separately.
Gamma-glutamyl transferase (GGT)	9 to 85 IU/L	Frequently elevated in MASLD, but it is not exclusively indicative of liver disease.
L-lactate dehydrogenase	0.4–1.7 µmol/L	Elevated levels are frequently indicative of liver disease.
Total bilirubin	2–21 µmol/L	Elevated concentrations indicate liver damage
Prothrombin time (PT)	25–41 s	Indicator of the status of blood clotting factor, and a longer time suggests a probable liver injury.
Albumin	3.5 to 5.3 g/dL	Albumin is a protein exclusively made by the liver, and low concentrations are indicative of impaired liver function.
Uric acid	M 2.1–8.5 mg/dL F 2.07–7.0 mg/dL	Elevated levels are believed to be highly predictive of steatotic liver disease.
Total bile acids	1–2 μg per mL	Moderate elevation and changes in bile acid compositions by changing farnesoid X receptor (FXR) activity in MASLD [22,23].
C-reactive protein (CRP)	<3 mg/dL	Inflammatory marker for MASLD [24].
Trimethylamine *N*-oxide (TMAO)	<6 μmol/L	TMA is produced by gut bacteria from dietary precursors (choline, L-carnitine, betaine) and quickly converted to TMAO in the liver. TMA is very low in the serum of a healthy person [25].
Fecal and serum butyrate	Not Assigned	Their concentrations are lower in MASLD patients by 20–50% than in healthy persons [26].

## Data Availability

Not applicable.

## Supplementary Materials

The following supporting information can be downloaded at: https://www.mdpi.com/article/10.3390/nu16183061/s1, Figure S1. Decision tree for safety assessment of health functional food ingredients provided by the Ministry of Korea Food and Drug Administration.

## Abbreviations

MASLD, Metabolic-Associated Steatotic Liver Disease; HFFs, Health functional foods; MRI, Magnetic Resonance Imaging; IR, Insulin Resistance; MetS, Metabolic Syndrome; ER, Endoplasmic Reticulum; MASH, Metabolic Dysfunction-Associated Steatohepatitis; PNPLA3, Patatin-Like Phospholipase Domain-Containing Protein 3; MFDA, Ministry of Food and Drug Administration; TG, Triglycerides; CoA, Coenzyme A; VLDL, Very Low-Density Lipoprotein; FFA, Free Fatty Acids; PSNS, Parasympathetic Nervous System; ALP, Alkaline Phosphatase; ALT, Alanine Transaminase; AST, Aspartate Transaminase; GGT, Gamma-Glutamyl Transferase; NLRP3, NOD-Like Receptor Protein 3; LC-MS/MS, Liquid Chromatography-Tandem Mass Spectrometry; UPLC-Q, Ultra-Performance Liquid Chromatography-Quadrupole; AKT1, AKT Serine/Threonine Kinase 1; IL6, Interleukin-6; VEGFA, Vascular Endothelial Growth Factor A; TNF, Tumor Necrosis Factor; PPARG, Peroxisome Proliferator-Activated Receptor Gamma; MAMP, Microbe-Associated Molecular Pattern; PAMP, Proadrenomedullin N-Terminal 20 Peptide; LPS, Lipopolysaccharides; TLR4, Toll-Like Receptor 4; PCB, Polychlorinated Biphenyl; SCFA, Short-Chain Fatty Acids; FXR, Farnesoid X Receptor; NF-κB, Nuclear Factor-Kappa B; SREBP-1c, Sterol Regulatory Element-Binding Protein 1c; GPBAR1, G Protein-Coupled Bile Acid Receptor 1; HDACs, Histone Deacetylases; GPCRs, G Protein-Coupled Receptors; FFAR-2, Free Fatty Acid Receptor 2; FFAR-3, Free Fatty Acid Receptor 3 (GPR41); TMA, Trimethylamine; TMAO, Trimethylamine N-Oxide; FMOs, Flavin-Containing Monooxygenases; 5-HT, Serotonin; MTT, Microbiome-Targeted Therapies; TNF-α, Tumor Necrosis Factor-Alpha; FMT, Fecal Microbiota Transplantation; ZO-1, Zonula Occludens-1; NR2F6, Nuclear Receptor Subfamily 2 Group F Member 6; circRNA, Circular RNA; AMPK, AMP-Dependent Protein Kinase; lncRHL, Long Non-Coding RNA Regulator of Hyperlipidemia; MTTP, Microsomal Triglyceride Transfer Protein.
