# Supplementary material for: Biomarkers for Health Functional Foods in Metabolic Dysfunction-Associated Steatotic Liver Disorder (MASLD) Prevention: An Integrative Analysis of Network Pharmacology, Gut Microbiota, and Multi-Omics
Source: Nutrients. 2024 Sep 11;16(18):3061. doi: 10.3390/nu16183061 (PMC11434757; doi:10.3390/nu16183061)
Supplement: Supplementary file 1 [file nutrients-16-03061-s001.zip › nutrients-3171508-supplementary.pdf]

Figure S1. Decision tree for safety assessment of health functional food ingredients provided by Korea Food & Drug Administration.

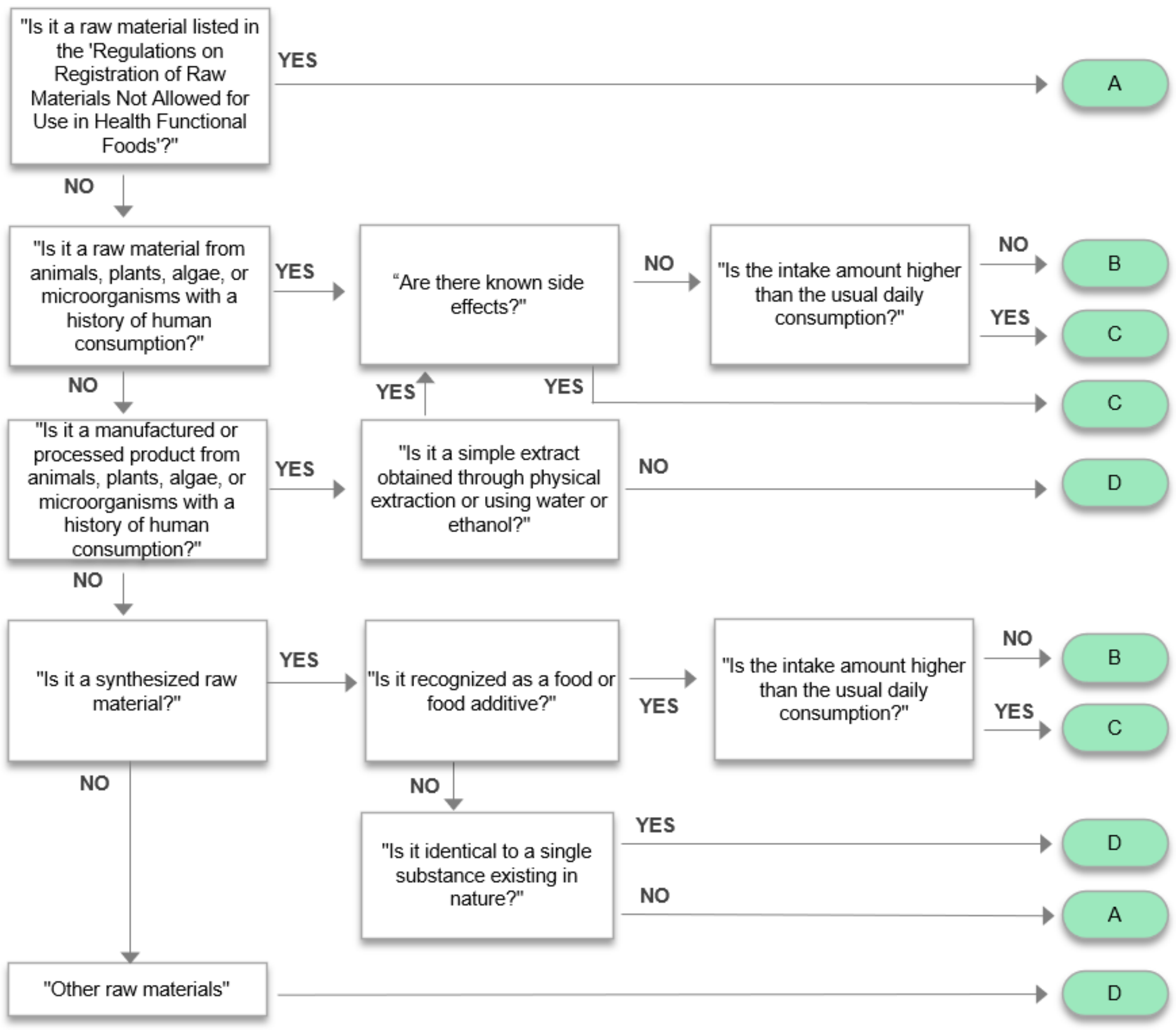

Safety data to be submitted.	A	B	C	D
Cannot be applied as a healthy health functional food.	√			
Evidence of consumption.		√	√	√
Safety information on the functional ingredient or related substances.		√	√	√
Intake evaluation data.		√	√	√
Nutritional evaluation data, biological availability data (or Bioavailability data), Human clinical trial data (or Human testing data)			√	√
Toxicity experimental data				√

A. Cannot be applied as a health functional food.

B. As evidence of prior consumption experience, materials such as the Health Functional Food Code, Food Code, Food Additive Code, scientific literature documenting traditional use, historical usage records, and recognition documents from foreign government agencies can be provided.

C. Data from database searches on the toxicity or safety of the functional ingredient or related substances.

D. Data comparing and analyzing the average intake and proposed intake based on the results of the National Nutrition Survey and other intake surveys.

E. Data evaluating whether the consumption of the raw material affects the absorption, distribution, metabolism, and excretion of other nutrients; bioavailability data; and human study data from intervention trials and epidemiological surveys.

F. Toxicity test data should be based on single-dose toxicity tests (in rodents and non-rodents), 3-month repeated-dose toxicity data (in rodents), and genetic toxicity tests (including the reverse mutation test, chromosomal aberration test, and micronucleus test). Depending on the characteristics of the raw material, additional tests for reproductive toxicity, antigenicity, immunotoxicity, and carcinogenicity may be required. However, exceptions can be made if other safety data can adequately demonstrate the safety of the material.
